# Prevalence and Clinical Implications of Post-obstruction Hyperdiuresis Among Patients with Urinary Retention: A Mini Review

**DOI:** 10.1016/j.euros.2025.01.017

**Published:** 2025-02-18

**Authors:** Ursina Rigonalli, Silvan Sigg, Seraina Von Moos, Philipp Baumeister, Agostino Mattei, Christian D. Fankhauser, Andres Affentranger

**Affiliations:** a Cantonal Hospital of Lucerne, Lucerne, Switzerland; b University of Lucerne, Lucerne, Switzerland; c University of Zurich, Zurich, Switzerland

**Keywords:** Urinary retention, Post-obstructive diuresis, Polyuria, Complication, Mini review

## Abstract

Urinary retention is a common urological emergency requiring catheterization. However, follow-up management remains poorly defined, particularly regarding post-obstruction hyperdiuresis (POHD), which may lead to complications such as hypovolemia and electrolyte disturbances. Our mini review of PODS identified nine relevant studies involving 665 patients. POHD occurred in 15–78% of cases, with a mean duration of 2–5 d. Risk factors included serum creatinine >105 μmol/l (odds ratio [OR] 4.83, 95% confidence interval [CI] 1.14–20.44; *p* = 0.032) and greater bladder volume (OR per 100-ml increment: 1.21, 95% CI 1.06–1.40; *p* = 0.006). Complications included hematuria (11–55%), hyponatremia (22–28%), and hypotension (9%), most of which were self-limiting. Data on management were sparse; one randomized controlled trial showed no significant difference in complications between rapid and gradual decompression. The lack of standardized protocols underscores the need for further prospective studies to optimize patient outcomes.

**Patient summary:**

After relief of urinary obstruction, an increase in urination is common. Complications such as blood in the urine, electrolyte imbalances, and dehydration may occur but typically resolve on their own without additional treatment.

## Take Home Message

Post-obstruction hyperdiuresis occurs in 15–78% of patients, with common but usually self-limiting complications such as hematuria, hyponatremia, and hypotension. Complication rates appear to be similar after rapid and gradual decompression methods.

## 1 Background

Urinary retention is a common urological emergency, affecting 2.2–6.8 per 1000 men annually [1]. Immediate management of urinary retention involves decompression of the bladder via urinary catheterization. However, bladder decompression can lead to potentially life-threatening post-obstruction hyperdiuresis (POHD), which is defined as a sustained urine output of at least 200 ml/h for 2 consecutive hours or a total urine output exceeding 3 l within 24 h [2]. In most patients, POHD resolves with the restoration of fluid and solvent homeostasis. In some cases, however, excessive excretion of water and solutes persists even after homeostasis is achieved. Several mechanisms are involved in the pathophysiology of PODS, including tubulopathy leading to lower sodium reabsorption, fluid overload due to acute renal failure, and various biochemical and immunological factors [2]. These processes place patients at significant risk of severe dehydration, electrolyte imbalances, hypovolemia, and potentially even death if the fluid and electrolyte disturbances are not adequately treated. The aim of our study was to review the incidence of complications including POHD following bladder decompression in patients with urinary retention (Table 1).

**Table 1:** Incidence of POHD and complications following bladder decompression

Study	Patients	Incidence, *n* (%)				
		POHD	Hematuria	Hypotension	Hyponatremia	IRF
Shapiro 2022 [4]	104	–	–	–	29 (28)	–
Leinum 2020 [8]	64	19 (29.7)	18 (28.1)	–	–	–
Boettcher 2013 [5]	294	–	32 (10.9)	0 (0)	–	–
Ahmed 2013 [9]	22	4 (18.2)	12 (54.5)	2 (9.1)	–	2 (9.1)
Goonewardena 2005 [10]	47	7 (14.9)	–	–	–	–
Jones 1988 [6]	21	–	–	–	–	–
O’Reilly 1986 [11]	36	28 (77.8)	–	–	–	–
Bishop 1985 [7]	55	29 (52.7)	–	–	12 (21.8)	–
Vaughan 1973 [3]	22	22 (100)	–	0 (0)	–	–
Overall (weighted mean)	665	44.2%	16.3%	**–**	**–**	**–**

POHD = post-obstruction hyperdiuresis; IRF = impaired renal function.

## 2 Methods

We conducted a systematic review of articles published in the PubMed database up to December 13, 2023. The search strategy is provided in the Supplementary material. Our analysis included studies that reported on the prevalence, risk factors, and management for POHD following urinary retention. To ensure comprehensive coverage of the relevant literature, we did not set predefined criteria for interventions, controls, or outcomes in our search strategy. We excluded case reports, systematic reviews, and editorials and only considered studies published in English. Two authors (U.R. and A.A.) independently screened the abstracts and full texts and performed data extraction. Any disagreements were resolved via consensus or, if necessary, third-party arbitration by a senior author (C.D.F.). A flowchart describing the study selection process is shown in Supplementary Fig. 1 and the risk-of-bias assessment is presented in Supplementary Table 3.

## 3 Results

We identified 1004 studies, of which 26 were selected for full-text review after title and abstract screening. Ultimately, nine studies published between 1973 and 2022 met the inclusion criteria for our analysis. These studies involved a total of 665 patients with a weighted mean age of 70.4 yr; the majority of the patients were male (93%, *n* = 616). Regarding the type of urinary retention, 31% of the patients (*n* = 208) had chronic urinary retention and 17% (*n* = 110) had acute urinary retention; no information on the type of urinary retention was available for 52% of the patients (*n* = 347). Benign prostatic enlargement was the most common cause of urinary retention (11%, *n* = 70), followed by prostate cancer (5%, *n* = 36). Other causes were urinary tract infection, urethral stricture, bladder cancer, and neurourological disorders.

The mean decompressed bladder volume among the studies ranged from 1 to 2.4 l, with a mean weighted volume of 1.3 l. The POHD incidence in the studies varied widely, ranging from 15% to 78% of patients. One study included only patients with POHD so the incidence in that study was 100% [3]. Three studies did not report the incidence of POHD [4], [5], [6]. The POHD duration was reported in two studies: the mean duration was 2 d in one study and 5 d in the other, and the total duration ranged from 1 d to 13 d [3], [7].

Data on patient characteristics before bladder decompression were limited. Impaired renal function, defined as plasma creatinine >120 μmol/l, was reported in five studies, with a weighted mean prevalence of 58% [7], [8], [9], [10], [11]. Notably, in one of these studies, complete recovery of impaired renal function was observed in 82% of patients affected after decompression [9]. Hypertension was another common comorbidity. The weighted mean incidence of hypertension among five studies was 39% [3], [4], [6], [7], [10]. In addition, one study described altered mental status in seven patients, all of whom recovered completely after bladder decompression [3]. Other clinical characteristics observed before decompression included peripheral edema, congestive heart failure, hyponatremia, and hyperkalemia. Most of these conditions were described as reversible after decompression.

The most common complication following bladder decompression was post-obstructive hematuria, with incidence ranging from 11% to 55%. In one study, all cases of hematuria resolved spontaneously within 24 h without the need for transfusion or surgical intervention [9]. Another study did not provide details on hematuria management [8], while a third study comparing rapid and gradual bladder decompression found similar hematuria rates between the two groups (11.3% vs 10.5%). Six patients in the rapid decompression group and four in the gradual decompression group required additional intervention, with no significant differences in the rate, severity, or timing of bleeding between the catheterization methods [5].

One study reported an incidence of post-obstructive hypotension in 9% (two patients); intravenous fluids were reserved for patients experiencing significant hemodynamic instability, while other cases were managed with oral hydration [9]. Two other studies reported no cases of post-obstructive hypotension [3], [5], and the hypotension incidence was not documented in the remaining studies.

Hyponatremia, defined as a sodium level <135 mmol/l, after catheterization was reported in two studies, with incidence rates of 22% (*n* = 12) [7] and 28% (*n* = 29) [4]. In one of these studies, 25% of the hyponatremic patients (*n* = 4) required sodium replacement therapy [7]. Conversely, another study observed spontaneous recovery of hyponatremia within 48 h in all patients affected, including those with severe hyponatremia (sodium <125 mmol/l). Management strategies included discontinuation of medications known to cause hyponatremia in patients with moderate to severe hyponatremia [4].

Several studies have discussed potential risk factors for POHD, but only one study used statistical tests for risk estimation [8]. The authors identified two independent risk factors via multivariate analysis: serum creatinine >105 μmol/l (odds ratio [OR] 4.83, 95% confidence interval [CI] 1.14–20.44, *p* = 0.032) and higher decompressed volume (OR per 100-ml increment: 1.21, 95% CI 1.06–1.40; *p* = 0.006). Other risk factors discussed but not statistically validated include hypertension, elevated serum urea, peripheral edema, congestive heart failure, and mental confusion [3], [8]. In addition, a randomized controlled trial found no significant difference in the complications rates between rapid and gradual bladder decompression [7].

## 4 Discussion

Urinary retention is a common urological emergency that is managed with bladder decompression. Our review indicates that POHD incidence varies widely, with rates ranging from 15% to 78%. Potential risk factors identified include elevated serum creatinine and higher urinary retention volume [8]. However, no studies have examined the type of urinary retention (acute vs chronic) as a potential risk factor. Considering high urinary volume as a proposed risk factor, patients with chronic urinary retention might be at higher risk. Nevertheless, owing to the limited data available, it remains unclear which patients are most likely to develop POHD.

The studies available provided limited data on the management of POHD and its associated complications. Only one study detailed a fluid replacement strategy, recommending replacement of 67% of urine output, with 65% administered intravenously and 35% orally [3]. None of the studies reviewed reported on intensive care unit admission rates or mortality rates associated with POHD. A randomized controlled trial comparing rapid versus gradual bladder decompression found no significant differences in the incidence of hematuria or hypotension following rapid decompression [5]. This suggests that the decompression method does not substantially affect complication rates.

Given the limited data on the prevalence, risk factors and management of post-obstruction hyperdiuresis, there is an urgent need for prospective cohort studies and standardized protocols to develop effective management strategies and improving patient outcomes in this critical area.

## 5 Conclusions

POHD occurs frequently following urinary retention, affecting between 15% and 78% of patients. Elevated serum creatinine and a larger bladder volume at decompression have been identified as significant risk factors. While complications such as hematuria (11–55%), hyponatremia (22–28%), and hypotension (9%) are common, they are typically self-limiting and resolve spontaneously. The method used for bladder decompression, whether rapid or gradual, does not significantly affect complication rates, suggesting that either approach can be safely used in clinical practice.

  ***Conflicts of interest:*** The authors have nothing to disclose.

  ***Use of generative AI and AI-assisted technologies:*** During the preparation of this manuscript the authors used OpenAI ChatGPT (September 2023 version) to enhance the writing and clarity of the text. After using this AI-assisted tool, the authors reviewed and edited the content as needed and take full responsibility for the content of the publication.
