# Supplementary material for: Prevalence and Clinical Implications of Post-obstruction Hyperdiuresis Among Patients with Urinary Retention: A Mini Review
Source: Eur Urol Open Sci. 2025 Feb 18;73:68–70. doi: 10.1016/j.euros.2025.01.017 (PMC11879703; doi:10.1016/j.euros.2025.01.017)
Supplement: Supplementary Appendix 1 [file mmc1.docx]

Prevalence and clinical implications of post-obstructive diuresis in patients with urinary retention, a mini review

Ursina Rigonalli^1,2^, Silvan Sigg^1,2^, Seraina Von Moos^1,2,3^, Philipp Baumeister^1,2^, Agostino Mattei^1,2^, Christian D. Fankhauser^1,2,3^, Andres Affentranger^1,2^

1. Luzerner Kantonsspital, Lucerne, Switzerland
2. University of Lucerne, Lucerne, Switzerland
3. University of Zurich, Zurich, Switzerland

**Literature search strategy for PubMed**

Population – Intervention – Control - Outcome

Evaluating the Incidence, Clinical Implications, and Safety of Outpatient Management of Polyuria and Electrolyte Imbalances Following Urinary Catheterization in Patients with Urinary Retention: A Systematic Review

Keywords and MeSH terms:

(

“Urinary Retention” [MeSH] OR (retention[title/abstract] AND urin*[title/abstract]) OR (bladder [Title/Abstract] AND retention [Title/Abstract])

)

AND

(

Polyuria [MeSH] OR polyuria [Title/Abstract] OR “increased urine output” [Title/Abstract] OR “excessive urin*” [Title/Abstract] OR “excess urine production” [Title/Abstract] OR

diuresis [Title/Abstract] OR

“Water-Electrolyte Imbalance” [MeSH] OR

(Electrolyt* [Title/Abstract] OR imbalance* [Title/Abstract])

)
