# Supplementary material for: Prevalence and Clinical Implications of Post-obstruction Hyperdiuresis Among Patients with Urinary Retention: A Mini Review
Source: Eur Urol Open Sci. 2025 Feb 18;73:68–70. doi: 10.1016/j.euros.2025.01.017 (PMC11879703; doi:10.1016/j.euros.2025.01.017)
Supplement: Supplementary Figure 1 [file mmc2.docx]

Studies from PubMed **(n = 1003)**

References from other sources **(n = 1)**

**Identification**

Studies included in review **(n = 9)**

Studies excluded **(n = 978)**

Studies not retrieved **(n = 0)**

Studies assessed for eligibility **(n = 26)**

Studies sought for retrieval **(n = 26)**

Studies screened **(n = 1004)**

Studies excluded **(n = 17)**

Review (n = 5)

Non-English (n = 1)

No full text available (n = 4)

Opinion or case report (n = 3)

Wrong outcomes (n = 3)

Wrong patient population (n = 1)

References removed **(n = 0)**

**Screening**

**Included**
