# Supplementary material for: Prevalence and Clinical Implications of Post-obstruction Hyperdiuresis Among Patients with Urinary Retention: A Mini Review
Source: Eur Urol Open Sci. 2025 Feb 18;73:68–70. doi: 10.1016/j.euros.2025.01.017 (PMC11879703; doi:10.1016/j.euros.2025.01.017)
Supplement: Supplementary Tables 1 and 2 [file mmc3.docx]

**Supplementary Table 1 Patient demographics and characteristics before bladder decompression**

| **Study** | **Year of publication** | **Number of patients** | **Male, n (%)** | **Age,**  **Years mean/median (range)** | **Decompressed volume,**  **mean liter (range)** | **Impaired renal function, n (%)** | **Hyper-tension,**  **n (%)** | **Peripheral edema,**  **n (%)** | **Hyponatremia,**  **n (%)** | **Mental confusion,**  **n (%)** | **Congestive heart failure,**  **n (%)** | **Elevated urea,**  **n (%)** |
| --- | --- | --- | --- | --- | --- | --- | --- | --- | --- | --- | --- | --- |
| Shapiro [1] | 2022 | 104 | 63 (61) | 84+/-8 | - | - | 78 (75) | - | 29 (27.9) | - | - | - |
| Leinum [2] | 2020 | 64 | 64 (100) | 72.5 (66-75) | 1 (0.6-1.5) | 22 (34.4) | - | - | - | - | - | - |
| Boettcher [3] | 2013 | 294 | 294 (100) | 72.5 (22-100) | 1.2 (0.2-4.1) | - | - | - | - | - | - | - |
| Ahmed [4] | 2013 | 22 | 22 (100) | 53 (18-78) | 1.5 (0.8-4.2) | 13 (59.1) | - | - | - | - | - | - |
| Goonewardena [5] | 2005 | 47 | 39 (83) | 56.3 (14-85) | 0.97 (0.4-2.3) | 20 (42.6) | 12 (25.5) | - | - | - | - | - |
| Jones [6] | 1988 | 21 | 21 (100) | 71 (34-87) | 1.1 | - | 11 (52.4) | 8 (38.1) | - | - | - | - |
| O’Reilly [7] | 1986 | 36 | 36 (100) | (47-89) | 2.4 (0.5-9.0) | 20 (55.6) | - | - | - | - | - | 20 (55.6) |
| Bishop [8] | 1985 | 55 | 55 (100) | 71 (52-89) | 1.4 (0.6-3.1) | 47 (85.5) | 27 (50) | 18 (32.7) | - | - | - | - |
| Vaughan [9] | 1973 | 22 | 22 (100) | 65.5 (34-85) | (1.0-3.5) | - | 17 (77.3) | 9 (40.9) | - | 7 (31.8) | 7 (31.8) | 16 (72.7) |
| **Total, weighted mean** | **-** | **665** | **616**  **(93)** | **70.4** | **1.27** | **54.5%** | **32.3%** | **-** | **-** | **-** | **-** | **-** |

*n: number*

**Supplementary Table 2 Underlying diagnosis for urinary retention**

| **Study** | **Type of retention, n (%)** | | | **Underlying diagnosis for urinary retention, n (%)** | | | | | | |
| --- | --- | --- | --- | --- | --- | --- | --- | --- | --- | --- |
|  | **chronic** | **acute** | **unknown** | **Benign prostatic enlargement** | **Prostate cancer** | **Prostatitis/Infection** | **Bladder cancer** | **Urethral stricture or valve** | **neurogenic** | **Other or unknown** |
| Shapiro [1] | 0  (0) | 104 (100) | 0  (0) | - | - | - | - | - | - | 104 (100) |
| Leinum [2] | 14 (21.8) | 0  (0) | 50  (78.1) | 24 (37.5) | 11 (17.2) | 6 (9.4) | 2 (3.1) | - | 6 (9.4) | 15 (23.4) |
| Boettcher [3] | 0  (0) | 0  (0) | 294  (100) | - | - | - | - | - | - | 294 (100) |
| Ahmed [4] | 22  (100) | 0  (0) | 0  (0) | 10 (45.5) | 1 (4.5) | - | 1 (4.5) | 8 (36.4)) | 2 (9.1) | 0 (0) |
| Goonewardena [5] | 47  (100) | 0  (0) | 0  (0) | 21 (44.7) | 5 (10.6) | 1 (2.1) | - | 6 (12.8) | 3 (6.4) | 16 (34.0) |
| Jones [6] | 21  (100) | 0  (0) | 0  (0) | - | - | - | - | - | - | 21 (100) |
| O’Reilly [7] | 36  (100) | 0  (0) | 0  (0) | - | 14 (38.9) | - | 5 (13.9) | - | - | 17 (36.2) |
| Bishop [8] | 55  (100) | 0  (0) | 0  (0) | - | - | - | - | - | - | 55 (100) |
| Vaughan [9] | 13  (59.1) | 6  (27.3) | 3  (13.6) | 15 (68.2) | 5 (22.7) | 2 (9.1) | - | 1 (4.5) | - | 0 (0) |
| **Total, n (%)** | **208 (31.3)** | **110 (16.5)** | **347**  **(52.2)** | **70 (10.5)** | **36 (5.4)** | **9 (1.4)** | **8 (1.2)** | **15 (2.3)** | **11 (1.7)** | **522 (80.5)** |

*n: number*

**References**

1. Shapiro, D.S., et al., *Prospective determination of the incidence and severity of hyponatraemia in older hospitalised patients with acute urinary tract obstruction.* Age Ageing, 2022. **51**(1).

2. Leinum, L.R., C. Berthelsen, and N. Azawi, *Post-obstructive diuresis; underlying causes and hospitalization.* Scandinavian Journal of Urology, 2020. **54**(3): p. 253-257.

3. Boettcher, S., et al., *Urinary Retention: Benefit of Gradual Bladder Decompression - Myth or Truth? A Randomized Controlled Trial.* Urologia Internationalis, 2013. **91**(2): p. 140-144.

4. Ahmed, M., et al., *Rapid and complete decompression of chronic urinary retention: a safe and effective practice.* Trop Doct, 2013. **43**(1): p. 13-6.

5. Goonewardena, S.A. and S. Sivapriyan, *High pressure chronic retention: a life-threatening clinical entity.* Ceylon Med J, 2005. **50**(2): p. 71-3.

6. Jones, D.A., et al., *The biphasic nature of renal functional recovery following relief of chronic obstructive uropathy.* Br J Urol, 1988. **61**(3): p. 192-7.

7. O'Reilly, P.H., et al., *High pressure chronic retention. Incidence, aetiology and sinister implications.* Br J Urol, 1986. **58**(6): p. 644-6.

8. Bishop, M.C., *Diuresis and renal functional recovery in chronic retention.* Br J Urol, 1985. **57**(1): p. 1-5.

9. Vaughan, E.D., Jr. and J.Y. Gillenwater, *Diagnosis, characterization and management of post-obstructive diuresis.* J Urol, 1973. **109**(2): p. 286-92.
