# Supplementary material for: Prevalence and Clinical Implications of Post-obstruction Hyperdiuresis Among Patients with Urinary Retention: A Mini Review
Source: Eur Urol Open Sci. 2025 Feb 18;73:68–70. doi: 10.1016/j.euros.2025.01.017 (PMC11879703; doi:10.1016/j.euros.2025.01.017)
Supplement: Supplementary Table 3 [file mmc4.docx]

**Supplementary Table 3 Risk of Bias Analysis**

| **Study** | **Bias Domain** | **Risk Level** | **Support for Judgment** |
| --- | --- | --- | --- |
| Shapiro [1] | (A) Bias arising from the randomization process | High | No randomization - prospective observational study with control group. Patients were assigned to groups based on whether they had urinary catheter placement for aUTO (n=104) or other reasons (n=100). |
|  | (B) Bias due to deviations from intended interventions | Low | Clear protocols for patient monitoring and data collection. Standard procedures for catheter insertion and sodium measurement. Consistent follow-up within 48 hours for all patients. |
|  | (C) Bias due to missing outcome data | Low | All patients were followed until discharge. Complete data collection for primary outcomes (sodium levels, hyponatremia). No patients lost to follow-up mentioned. |
|  | (D) Bias in measurement of the outcome | Low | Primary outcomes were objective laboratory measurements (serum sodium levels). Standardized definitions used for hyponatremia (<135 mEq/l) and severe hyponatremia (<125 mEq/l). |
|  | (E) Bias in selection of the reported results | Low | All pre-specified outcomes reported with appropriate statistical analyses. Complete reporting of baseline characteristics and outcomes for both groups. Well-documented comparison of hyponatremia rates. |
|  | (F) Overall bias | Some concerns | While outcome measurement and follow-up were strong, the non-randomized design could introduce selection bias. However, baseline characteristics were similar between groups, and the prospective design strengthens the findings. |
| Leinum [2] | (A) Bias arising from the randomization process | High | This was a retrospective chart review study with no randomization. Patient data was collected from consecutive cases meeting inclusion criteria. |
|  | (B) Bias due to deviations from intended interventions | Low | The study followed a clear protocol for data collection using Sarkar and Seshadri's six steps for chart review and the RECORD checklist. Data collection procedures were standardized and well-documented. |
|  | (C) Bias due to missing outcome data | Some concerns | Of 155 identified patients, 44 were excluded due to ineligibility or missing data. Additional analyses on 40 of 64 patients were performed due to incomplete data. Authors acknowledge incomplete nursing observations. |
|  | (D) Bias in measurement of the outcome | Some concerns | While laboratory values were objective, POD definition criteria may have some subjectivity. No mention of whether chart reviewers were blinded. Authors used a standardized data extraction instrument. |
|  | (E) Bias in selection of the reported results | Low | Study followed RECORD guidelines for reporting. All pre-specified outcomes were reported with appropriate statistical analyses. Results include both positive and negative findings. |
|  | (F) Overall bias | High | Primary concerns are the retrospective design, significant missing data, and potential measurement bias. However, the methodology was transparent and followed reporting guidelines. |
| Boettcher [3] | (A) Bias arising from the randomization process | Some concerns | Quote: "Patients were randomized to RD or GD by drawing lots." Method of lot drawing and allocation concealment not described. Baseline characteristics were balanced between groups. |
|  | (B) Bias due to deviations from intended interventions | Low risk | Protocol appears to have been followed as planned. The nature of interventions (GD vs RD) makes it unlikely there were deviations. Treatment protocols were clearly defined (200ml intervals for GD, continuous drainage for RD). |
|  | (C) Bias due to missing outcome data | Low risk | Only 20 out of 314 patients (6.4%) excluded due to missing data. Balanced between groups (final analysis: 142 GD vs 152 RD). Small proportion of missing data unlikely to significantly impact results. |
|  | (D) Bias in measurement of the outcome | Some concerns | No mention of blinding of outcome assessors. While some outcomes were objective (blood pressure, heart rate), assessment of hematuria severity could be subjective. Standardized monitoring protocol was used for all patients. |
|  | (E) Bias in selection of the reported results | Low risk | All pre-specified outcomes in methods section were reported in results. Appropriate statistical methods used. Results reported completely with relevant measures of precision. |
|  | (F) Overall bias | Some concerns | Main concerns arise from unclear randomization process and potential measurement bias due to lack of blinding. However, objective outcomes and balanced groups suggest results are likely reliable. |
| Ahmed [4] | (A) Bias arising from the randomization process | High | No randomization - this was a prospective observational study without a control group. All consecutive patients received the same intervention (rapid decompression). |
|  | (B) Bias due to deviations from intended interventions | Low | The intervention protocol was clearly defined and appears to have been consistently applied. All patients received rapid and complete decompression according to the described protocol. |
|  | (C) Bias due to missing outcome data | Low | All 22 patients who met inclusion criteria were followed and analyzed. No participants were lost to follow-up during the observation period. |
|  | (D) Bias in measurement of the outcome | Some concerns | While some outcomes were objective (urine output, blood pressure), the assessment criteria for some complications (e.g., severity of hematuria) were not clearly defined. No mention of blinded outcome assessment. |
|  | (E) Bias in selection of the reported results | Low | Study outcomes were clearly pre-specified and reported completely. Results include both positive and negative findings. Appropriate reporting of all relevant clinical outcomes. |
|  | (F) Overall bias | High | Primary concern is the non-randomized, single-arm design without a control group. While execution appears sound, the study design limits ability to draw comparative conclusions about safety and efficacy. |
| Goonewardena [5] | (A) Bias arising from the randomization process | High | No randomization - prospective descriptive study of consecutive patients. Selection based on specific clinical criteria for HPCR. No control group. |
|  | (B) Bias due to deviations from intended interventions | Some concerns | While diagnostic criteria were clear, lack of urodynamic evaluation facilities was noted as a drawback. Protocol for patient monitoring and treatment not fully detailed. |
|  | (C) Bias due to missing outcome data | Some concerns | Ultrasonography performed in 44 out of 47 patients (missing data for 3 patients). Reason for missing ultrasounds not explained. Otherwise complete follow-up for included patients. |
|  | (D) Bias in measurement of the outcome | Some concerns | Clear diagnostic criteria used but some key measurements (e.g., urodynamics) were unavailable. Standardized definitions provided for HPCR and post-obstructive diuresis, but assessment methods not fully detailed. |
|  | (E) Bias in selection of the reported results | Low | Comprehensive reporting of pre-specified outcomes. Clear presentation of patient characteristics and findings in tables. Both positive and negative outcomes reported. |
|  | (F) Overall bias | High | While the descriptive data is valuable, multiple methodological limitations exist including lack of control group, incomplete diagnostic testing, and some missing data. Study provides important observational data but causal inferences limited. |
| Jones [6] | (A) Bias arising from the randomization process | High | No randomization - prospective observational study of consecutive patients with HPCR. No control group. Single-arm study design. |
|  | (B) Bias due to deviations from intended interventions | Low | Clear, standardized protocols for measurements and monitoring. Multiple validated methods used to measure GFR (99mTc-DTPA and iohexol clearance). Consistent follow-up schedule for all patients. |
|  | (C) Bias due to missing outcome data | Low | Complete follow-up data for all 21 patients through 3 months. Comprehensive data collection at all specified time points. Only 2 patients excluded from IVP due to high creatinine (with clear explanation). |
|  | (D) Bias in measurement of the outcome | Low | Multiple objective outcome measures used (GFR, electrolytes, creatinine). Standardized collection protocols. Clear definitions of measurement methods. Close supervision of 24h urine collections by named investigator. |
|  | (E) Bias in selection of the reported results | Low | All pre-specified outcomes reported comprehensively. Clear presentation of both positive and negative findings. Complete data reporting in tables and figures with confidence intervals. |
|  | (F) Overall bias | Some concerns | While methodology is robust with excellent outcome measurement and follow-up, the non-randomized single-arm design limits causal inference. However, the prospective nature and comprehensive data collection strengthen findings. |
| O’Reilly [7] | (A) Bias arising from the randomization process | High | No randomization - prospective observational study of consecutive HPCR cases. No control group for primary outcomes, though comparison made with general prostatectomy population for malignancy rates. |
|  | (B) Bias due to deviations from intended interventions | Low | Clear diagnostic criteria and standardized protocols. Consistent approach to patient management based on predefined criteria (e.g., catheterization if urea >20 mmol/l). Standard surgical treatment for all patients. |
|  | (C) Bias due to missing outcome data | Low | Complete follow-up data reported for all 36 patients. All patients underwent planned interventions and assessments. No apparent loss to follow-up. |
|  | (D) Bias in measurement of the outcome | Low | Objective outcome measures used (urea, creatinine, urography findings, histology). Clear diagnostic criteria. Standardized measurement protocols. Histological confirmation of malignancy. |
|  | (E) Bias in selection of the reported results | Low | Comprehensive reporting of all pre-specified outcomes. Complete data presentation including negative findings. Appropriate statistical analysis with significance levels reported. |
|  | (F) Overall bias | Some concerns | While methodology is strong with complete follow-up and standardized measurements, the non-randomized design limits causal inference. However, the prospective nature and complete case capture strengthen findings for incidence estimates. |
| Bishop [8] | (A) Bias arising from the randomization process | High | No randomization - prospective observational study without control group. Patients were consecutively selected based on having chronic urinary retention with renal failure or upper tract dilatation. |
|  | (B) Bias due to deviations from intended interventions | Low | Clear protocol for patient monitoring with standardized measurements including daily weights, blood pressure, plasma creatinine, sodium, potassium and 24-hour urine volume. Consistent intervention (catheterization) and follow-up procedures. |
|  | (C) Bias due to missing outcome data | Low | Complete follow-up data for all 55 patients. Clear documentation of outcomes including renal function, diuresis, and clinical parameters. Study provides detailed data tables and figures showing outcomes for all participants. |
|  | (D) Bias in measurement of the outcome | Low | Used objective outcome measures (laboratory values, urine output, blood pressure). Clear definitions provided for key outcomes (e.g., diuresis defined as >3L/24h). Standardized monitoring protocols used. |
|  | (E) Bias in selection of the reported results | Low | Comprehensive reporting of all pre-specified outcomes. Results include both positive and negative findings. Clear presentation of data through tables and figures. Study limitations acknowledged. |
|  | (F) Overall bias | Some concerns | While methodology and reporting are robust, the non-randomized single-arm design limits causal inference. However, the prospective nature, complete follow-up, and objective outcomes strengthen the findings. |
| Vaughan [9] | (A) Bias arising from the randomization process | High | No randomization - prospective observational study of consecutive patients meeting specific diuresis criteria (>200ml/hr for 12 consecutive hours). No control group. |
|  | (B) Bias due to deviations from intended interventions | Low | Clear protocol for patient monitoring and data collection. Standardized criteria for measurements and interventions (ADH/DOCA tests). Consistent follow-up procedures described. |
|  | (C) Bias due to missing outcome data | Low | Complete data collection and follow-up for all 22 patients who met inclusion criteria. Clear documentation of clinical course and outcomes. |
|  | (D) Bias in measurement of the outcome | Low | Objective outcome measures used (urine output, electrolytes, blood pressure, weight). Standardized protocols for measuring responses to ADH and DOCA. Clear documentation of clinical parameters. |
|  | (E) Bias in selection of the reported results | Low | Comprehensive reporting of all pre-specified outcomes. Results presented for all patients, including both positive and negative findings. Detailed clinical data provided in tables. |
|  | (F) Overall bias | Some concerns | While methodology and reporting are strong, the non-randomized design and lack of control group limit causal inference. However, the prospective nature and standardized protocols strengthen the findings. |

**References**

1. Shapiro, D.S., et al., *Prospective determination of the incidence and severity of hyponatraemia in older hospitalised patients with acute urinary tract obstruction.* Age Ageing, 2022. **51**(1).

2. Leinum, L.R., C. Berthelsen, and N. Azawi, *Post-obstructive diuresis; underlying causes and hospitalization.* Scandinavian Journal of Urology, 2020. **54**(3): p. 253-257.

3. Boettcher, S., et al., *Urinary Retention: Benefit of Gradual Bladder Decompression - Myth or Truth? A Randomized Controlled Trial.* Urologia Internationalis, 2013. **91**(2): p. 140-144.

4. Ahmed, M., et al., *Rapid and complete decompression of chronic urinary retention: a safe and effective practice.* Trop Doct, 2013. **43**(1): p. 13-6.

5. Goonewardena, S.A. and S. Sivapriyan, *High pressure chronic retention: a life-threatening clinical entity.* Ceylon Med J, 2005. **50**(2): p. 71-3.

6. Jones, D.A., et al., *The biphasic nature of renal functional recovery following relief of chronic obstructive uropathy.* Br J Urol, 1988. **61**(3): p. 192-7.

7. O'Reilly, P.H., et al., *High pressure chronic retention. Incidence, aetiology and sinister implications.* Br J Urol, 1986. **58**(6): p. 644-6.

8. Bishop, M.C., *Diuresis and renal functional recovery in chronic retention.* Br J Urol, 1985. **57**(1): p. 1-5.

9. Vaughan, E.D., Jr. and J.Y. Gillenwater, *Diagnosis, characterization and management of post-obstructive diuresis.* J Urol, 1973. **109**(2): p. 286-92.
